# MCM8 variants in two patients with primary ovarian insufficiency: clinical findings and *in vitro* defective DNA repair revealed by an MCM8 double-mutant construct

**DOI:** 10.3389/fendo.2026.1807887

**Published:** 2026-06-17

**Authors:** Fei Wang, Shaolian Zang, Pin Li, Xiaoqin Yin

**Affiliations:** 1 Department of Endocrinology, Shanghai Children’s Hospital, School of Medicine, Shanghai Jiao Tong University, Shanghai, China; 2 Department of Urology, Shanghai Children’s Hospital, School of Medicine, Shanghai Jiao Tong University, Shanghai, China

**Keywords:** apoptosis, cell cycle, DNA repair, minichromosome maintenance complex component 8 (MCM8), primary ovarian insufficiency

## Abstract

**Backgrounds:**

Pediatric-onset primary ovarian insufficiency (POI) presents distinct clinical challenges, including delayed puberty or growth retardation. Previously, we reported a Chinese family with POI harboring compound heterozygous variants (p.C242R and p.S445*) in *MCM8* gene, a critical factor for DNA repair and gonadal homeostasis.

**Objective:**

This study describes clinical findings of pediatric-onset POI patients with compound heterozygous *MCM8* variants and investigates the *in vitro* functional consequences of an artificial double-mutant MCM8 construct harboring both p.C242R and p.S445*. Using this overexpression model, we assessed DNA damage response, protein interactions, and transcriptomic changes associated with the double-mutant MCM8.

**Methods:**

HeLa cell models were used to overexpress double-mutant (p.C242R and p.S445*) or wild-type MCM8. Functional consequences were evaluated using immunofluorescence staining, flow cytometry, and western blotting to assess DNA damage repair, cell cycle and apoptosis. Co-immunoprecipitation coupled with mass spectrometry (CoIP–MS/MS) was used to screen the disrupted protein interactome. Cut&Tag and mRNA-seq analyses were used to examine chromatin-binding-associated changes and transcriptomic alterations.

**Results:**

Ovarian tissues from POI patients exhibited reduced MCM8 expression and increased apoptosis. Cells expressing the artificial double-mutant MCM8 construct showed delayed resolution of DNA damage, as indicated by persistent γ-H2AX accumulation, and altered recruitment of DMC1 and RAD51. These changes were accompanied by S-phase accumulation and increased apoptosis. CoIP–MS/MS analysis identified altered interaction between double-mutant MCM8 and MCM6, suggesting possible changes in replication-associated protein interactions. Integrated CUT&Tag and RNA-seq analyses identified MCM8-associated chromatin-binding changes and transcriptomic alterations involving candidate genes enriched in ovarian survival and metabolic pathways, including the PI3K/AKT signaling pathway.

**Conclusions:**

These specific *MCM8* variants are clinically associated with pediatric-onset POI. *In vitro*, expression of double-mutant MCM8 construct was associated with impaired DNA damage resolution, altered MCM8-associated protein interactions, transcriptomic alterations, cell-cycle disturbance, and increased apoptosis. These findings provide cellular observations that may help generate hypotheses regarding how MCM8 dysfunction could contribute to ovarian insufficiency.

## 1 Introduction

Primary ovarian insufficiency (POI) is a clinical condition characterized by the cessation of ovarian function before the age of 40 and presents as amenorrhea, decreased estrogen levels, and elevated gonadotropin concentrations (1, 2). The diagnostic criteria for POI, according to the guidelines of the European Society of Human Reproduction and Embryology (ESHRE) (3, 4) and the National Institute for Health and Care Excellence (NICE) (5), include menstrual disturbance (such as primary amenorrhea, oligomenorrhea, or secondary amenorrhea (SA) for at least 4 months) and elevated follicle-stimulating hormone (FSH) levels exceeding 25 IU/L or 30 IU/L, respectively, measured on 2 different occasions at least 4 weeks apart. The same diagnostic principles are applied in adolescents (6). The etiology of pediatric POI is multifactorial and complex, encompassing genetic predispositions, autoimmune disorders, iatrogenic factors (such as chemotherapy and radiation therapy), and environmental influences (1, 7–9). Genome-wide association studies (GWASs) have revealed genetic variants associated with POI, which may manifest as ovarian dysfunction as early as childhood (10, 11). The clinical manifestations of pediatric POI are complex and heterogeneous and often exhibit considerable variation because of individual differences. Early identification and accurate diagnosis are critical for improving long-term prognostic outcomes in affected patients.

Minichromosome maintenance complex component 8 (MCM8), a member of the minichromosome maintenance (MCM) protein family, is associated with POI and plays a critical role in ovarian development during prenatal fetal ovarian development (12, 13). However, the underlying molecular mechanisms remain poorly understood. MCM8 is located on chromosome 20p12.3 and comprises 19 exons that encode a protein of 840 amino acids (14). This protein contains an N-terminal DNA-binding domain, including a zinc finger motif, as well as a C-terminal AAA+ core domain, and functions as a DNA helicase involved in DNA replication, meiosis, and homologous recombination (HR) (15, 16). In particular, MCM8 plays a crucial role in meiotic prophase during prenatal fetal ovarian development (17, 18). MCM8 not only is involved in DNA replication but also serves as a novel regulator of germ cell survival. Upon the occurrence of a DNA double-strand break (DSB), MCM8 activates the cellular DNA damage response (DDR) pathway, leading to rapid phosphorylation of histone H2AX (γ-H2AX) (19, 20). Simultaneously, upon the induction of double-strand breaks (DSBs), MCM8 expression is promptly upregulated, and the protein is recruited to DNA damage sites. It associates with MCM9 to form a complex demonstrating intrinsic DNA helicase activity, which synergistically modulates MCM9 stability and enables the recruitment of the MRE11–RAD50–NBS1 (MRN) complex to the break site during HR (21, 22). This complex subsequently orchestrates the assembly of downstream effector proteins, including recombinases, thereby increasing RAD51 loading onto single-stranded DNA and contributing to the stabilization and protection of genomic integrity (23, 24). MCM6 is a core component of the classic MCM2–7 complex, forming the catalytic core of the helicase for DNA replication in eukaryotes. It is loaded onto the replication origin during the G1 phase and drives DNA synthesis in the S phase (25). The knockdown of MCM6 can lead to delayed progression in the S/G2 phase, inhibiting cell proliferation, migration and invasion (26). The replication process driven by MCM6 may rely on the repair mechanism mediated by MCM8/9 to maintain genomic integrity (24).

In our previous study, we reported a Chinese family with pediatric-onset POI that harbors two compound heterozygous variants in *MCM8* gene (NM_001281522.1: c.724T>C [p.Cys242Arg] and c.1334C>A [p.Ser445*]). These variants were classified as pathogenic according to ACMG criteria. Because MCM8 is implicated in DNA repair and gonadal maintenance, these variants may be clinically relevant to the POI phenotype. However, the cellular consequences of this specific variant combination remain insufficiently characterized. In the present study, we describe the clinical and histological findings of the affected patients and explore the *in vitro* cellular effects using an artificial cis double-mutant MCM8 construct harboring both p.C242R and p.S445*. This *in vitro* model was used to assess the combined effects of the two variants under controlled experimental conditions and was not intended to reproduce the patients’ physiological trans compound heterozygous genotype. This study may provide cellular observations which could help generate hypotheses regarding how MCM8 dysfunction could contribute to ovarian insufficiency.

## 2 Materials and methods

### 2.1 Participants

In this study, a Chinese family with two compound heterozygous variants in the *MCM8* gene was recruited. The variants were identified by whole-exome sequencing (WES) and were confirmed in her family members and matched controls by Sanger sequencing. The proband was a 13-year-old girl who presented with delayed puberty, ovarian insufficiency, short stature, significantly elevated levels of luteinizing hormone (LH) and follicle-stimulating hormone (FSH)—consistent with hypergonadotropic hypogonadism—and low anti-Müllerian hormone (AMH) levels. The proband’s younger sister was 6 years and 7 months of age and was assessed by genetic testing when the uterine and ovarian volumes and the FSH level were normal, but unexpectedly, at the age of 7 years and 10 months, ultrasound revealed that the bilateral ovaries gradually progressed from a normal size to an unclear display, accompanied by a decrease in the FSH level. At follow-up at 11 years of age, the FSH levels progressively manifested as hypergonadotrophic hypogonadism. All tissue acquisitions were conducted during surgical diagnosis, and informed consent was obtained from the guardians of the patients and the subjects in the control group. Control ovarian tissue was obtained from adolescent girls who had undergone surgical removal due to ovarian torsion. Ovarian tissue from the proband was subjected to histopathological examination, immunofluorescence staining, and TUNEL-based apoptosis analysis. This study received approval from the Ethics Committee of Shanghai Children’s Hospital (2024R051-E01), and written informed consent was obtained from the individual and legal guardian.

### 2.2 Cell culture

HeLa cells, a human cervical carcinoma cell line, were obtained from the Cell Bank of the Chinese Academy of Sciences. The cells were maintained in DMEM supplemented with 10% fetal bovine serum, 100 U/mL penicillin, and 100 μg/mL streptomycin. The cultures were incubated at 37 °C under a humidified atmosphere of 5% CO_2_.

### 2.3 Western blotting

Proteins were extracted using lysis buffer (Thermo, MI, USA) supplemented with protease inhibitor (Roche, Japan). After quantification with a BCA Protein Assay Kit (Thermo, MI, USA), 20 μg of protein lysate was loaded and separated by sodium dodecyl sulfate–polyacrylamide gel electrophoresis (SDS–PAGE). The proteins were then transferred to polyvinylidene fluoride (PVDF) (Millipore, USA) membranes and incubated with primary antibodies overnight at 4 °C. Following washing, the membranes were incubated with HRP-conjugated secondary antibodies for 1 hour at room temperature. Protein signals were detected using an enhanced chemiluminescence (ECL) system, and protein intensities were quantified using ImageJ software. A complete list of antibodies used in this study is provided in **Supplementary Table 1**.

### 2.4 Plasmid construction and cell transfection

Wild-type and mutant eukaryotic expression vectors for the *MCM8* gene were constructed and packaged by OBiO Technology (Shanghai, China). A single MCM8 expression plasmid harboring both the c.724T>C (p.C242R) and c.1334C>A (p.S445*) variants was constructed. All plasmids were confirmed by Sanger sequencing. The plasmids were amplified and purified using a plasmid extraction kit (TIANGEN, China). To evaluate the functional impact of the *MCM8* variants, cells were seeded into 6-well plates at a density of 5 × 10^5^ cells per well. Cells were transiently transfected with plasmids encoding the wild-type MCM8, the p.Cys242Arg (C242R) variant, the p.Ser445* (S445*) variant, or both the c.724T>C (p.C242R) and c.1334C>A (p.S445*) variants using Lipofectamine™ 3000 reagent (Thermo Fisher, USA) according to the manufacturer’s instructions and incubated for 48 hours post-transfection. At 12 hours after transfection, the culture medium was replaced. To induce DNA double-strand breaks (DSBs), transfected cells were treated with 600 nM mitomycin C (MMC) (GLPBIO, USA) for 21 hours. Following MMC treatment, the cells were either harvested immediately or allowed to recover in fresh complete medium at 37 °C for 2 hours prior to collection for downstream analyses.

### 2.5 Immunofluorescence staining

Immunofluorescence staining was performed to assess the expression levels of MCM8 and to evaluate apoptosis in MCM8-mutant ovarian tissue from patients with POI. The tissues were incubated with primary antibodies for 24 hours at 4 °C. Following the washing steps, the corresponding fluorescently labeled secondary antibodies were applied and incubated for 1 hour in the dark. 4’,6-Diamidino-2-phenylindole (DAPI) and anti-fade mounting medium were subsequently added to the samples. Finally, fluorescence images were captured and analyzed using a fluorescence microscope (Leica, Germany). Detailed information on the antibodies used in the study is provided in **Supplementary Table 1**.

### 2.6 Immunohistochemical staining

Formalin-fixed, paraffin-embedded (FFPE) tissue sections (4 µm thick) were subjected to antigen retrieval in citrate buffer at 95 °C for 20 minutes. Endogenous peroxidase activity was blocked by incubation with 3% H_2_O_2_ for 15 minutes at room temperature. Following blocking with 5% normal serum for 1 hour at room temperature, the sections were incubated overnight at 4 °C with primary antibodies. Horseradish peroxidase (HRP)-conjugated secondary antibodies were then applied for 1 hour at room temperature. Immunoreactivity was visualized using 3,3′-diaminobenzidine (DAB) as the chromogen, followed by hematoxylin counterstaining. Slides were imaged using an Olympus BX53 microscope and independently scored by two pathologists who were blinded to the experimental conditions on the basis of a semiquantitative scoring system (0–3 scale).

### 2.7 Coimmunoprecipitation

Cells were lysed in NP-40 lysis buffer (a nonionic detergent) supplemented with protease inhibitors. The lysates were precleared using Protein A/G beads for 1 hour at 4 °C, followed by incubation of 500 µg of protein with 2 µg of specific antibody or control IgG overnight at 4 °C. Immune complexes were then captured with Protein A/G beads for 4 hours, after which they were washed four times with lysis buffer and eluted in Laemmli buffer. Coprecipitated proteins were subsequently analyzed by immunoblotting and mass spectrometry.

### 2.8 Cell cycle analysis

To analyze the cell cycle, the cells were fixed in ice-cold 70% ethanol for at least 2 hours. Following fixation, the cells were treated with RNase A (100 μg/mL; Thermo Fisher, USA) to digest the RNA and then stained with propidium iodide (50 μg/mL; Thermo Fisher, USA) for DNA content analysis. The samples were analyzed using a BD FACS flow cytometer (BD Biosciences, USA), and cell doublets were excluded by plotting the forward scatter width (FSC-W) against the forward scatter area (FSC-A). The proportions of cells in the G0/G1, S, and G2/M phases were determined using FlowJo software, and the sub-G1 peak was used as an indicator of apoptotic cells.

### 2.9 Cleavage under targets and tagmentation assay

To map the genome-wide chromatin occupancy of MCM8, we performed a Cut&Tag assay (GenSeq^®^ Cut&Tag Kit, GenSeq Inc.) according to established protocols with minor modifications. In brief, nuclei from HeLa cells expressing wild-type (WT) MCM8 and double-mutant (C242R-S445*) MCM8 were isolated, permeabilized, and immobilized on concanavalin A-coated magnetic beads. The chromatin was then sequentially incubated with a primary anti-MCM8 antibody, a secondary antibody, and a protein A-Tn5 transposase fusion protein preloaded with sequencing adapters. After activation with Mg²^+^ to initiate targeted tagmentation, DNA was purified to generate sequencing libraries using GenSeq^®^ 2× HiFi PCR Mix (GenSeq Inc.). A control sample using rabbit IgG was processed in parallel. The amplified libraries were quality-checked by Agilent 2100 Bioanalyzer and then subjected to high-throughput sequencing on Illumina NovaSeq sequencing platform (Illumina Novaseq 6000). Using bowtie2 software (v2.2.4) align high-quality reads onto the reference genome. MACS software (v1.4.2) was used for Peak Calling, diffReps software (v1.55.4) was used to identify differentially enriched regions, and UCSC RefSeq database was used for annotation to link peak and gene information. Differential binding analysis was performed to identify sites with significantly altered MCM8 occupancy in mutant versus WT cells.

### 2.10 Statistical analysis

All the quantitative data are expressed as the mean ± standard deviation (SD) of a minimum of three biological replicates. Statistical significance between experimental groups was determined by applying an unpaired, two-tailed Student’s t test using IBM SPSS Statistics (version 13.0). The data met the assumptions of the t test (normal distribution and homogeneity of variance). One-way ANOVA with Tukey’s *post hoc* test was used for multigroup comparisons. The associated candidate genes detected by Cut&Tag sequencing (fold change > 1) and the differentially expressed genes detected by mRNA-seq (fold change >= 1.5 and adjusted *P*-value [FDR] < 0.05) were intersected to identify genes associated with both altered chromatin binding and transcriptomic changes after expression of double-mutant MCM8 construct. Differences were considered statistically significant at P < 0.05. Graphical representations of the data were created with GraphPad Prism (version 8.0.1).

## 3 Results

### 3.1 Clinical and histological findings in patients carrying compound heterozygous MCM8 variants

**Figure 1:**
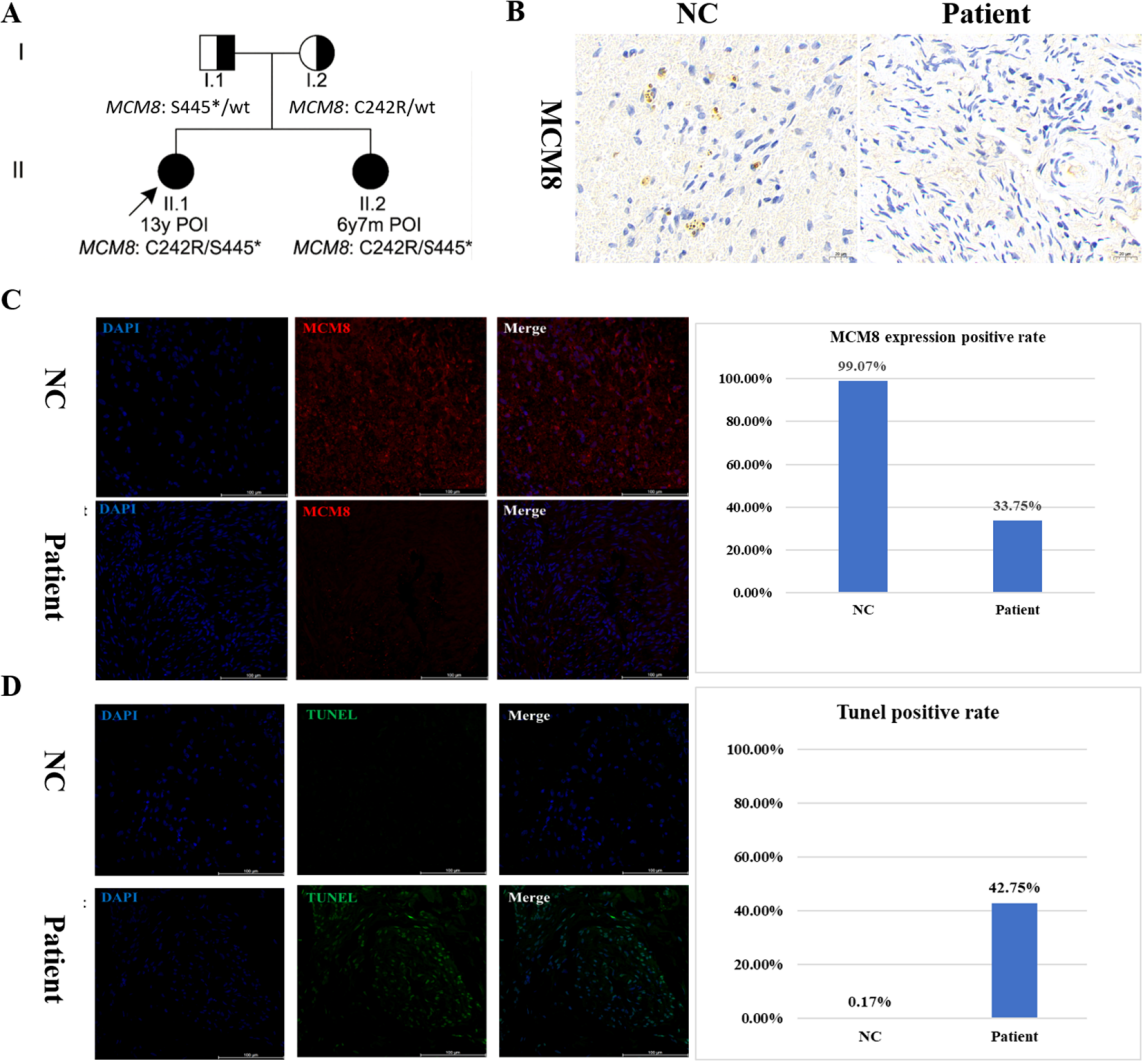
Clinical and histological findings in patients carrying compound heterozygous MCM8 variants. **(A)** Pedigree of the family carrying *MCM8* variants. The proband is Subject II-1 (arrow). **(B)** Immunohistochemical staining of MCM8 in ovarian tissue from the proband and control ovarian tissue. Hematoxylin stains the cell nucleus blue, and positive expression of MCM8 appears as brownish yellow. **(C)** Immunofluorescence staining and quantification of MCM8-positive cells in ovarian tissue. DAPI-stained cell nuclei appear blue, and positive expression of MCM8 with corresponding fluorophore labeling emits a red light. **(D)** Fluorescence images and positive rates of TUNEL staining in the ovarian tissues of patients with MCM8 variants. NC: normal ovarian tissue. Patient: Patient with MCM8 mutation. DAPI stains the cell nuclei blue. TUNEL fluorescein labeling appears as a green light.

Pedigree analysis showed that the father carried a heterozygous *MCM8* p.C242R variant, whereas the mother carried a heterozygous *MCM8* p.S445* variant. Both affected daughters inherited the two variants and carried compound heterozygous *MCM8* variants, p.C242R and p.S445* (**Figure 1A**). The proband was diagnosed with POI at 13 years of age, and her younger sister was diagnosed with POI at 6 years and 7 months of age. These findings support the clinical association between biallelic *MCM8* variants and pediatric-onset POI in this family. Compared with control ovarian tissue, patient-derived streak gonadal tissue showed reduced MCM8 staining (**Figure 1B**). Quantitative analysis showed that the percentage of MCM8-positive cells was lower in the proband**’**s tissue (33.75%) than in the control (99.07%) (**Figure 1C**). TUNEL staining showed increased apoptosis in patient-derived tissue, with TUNEL-positive cells accounting for 42.75% of cells in the proband**’**s tissue and 0.17% in control (**Figure 1D**). These findings indicate reduced MCM8 expression and increased apoptosis in patient-derived streak gonadal tissue, and they are presented as descriptive histological observations.

### 3.2 DNA damage and repair abnormalities associated with double-mutant MCM8 construct

**Figure 2:**
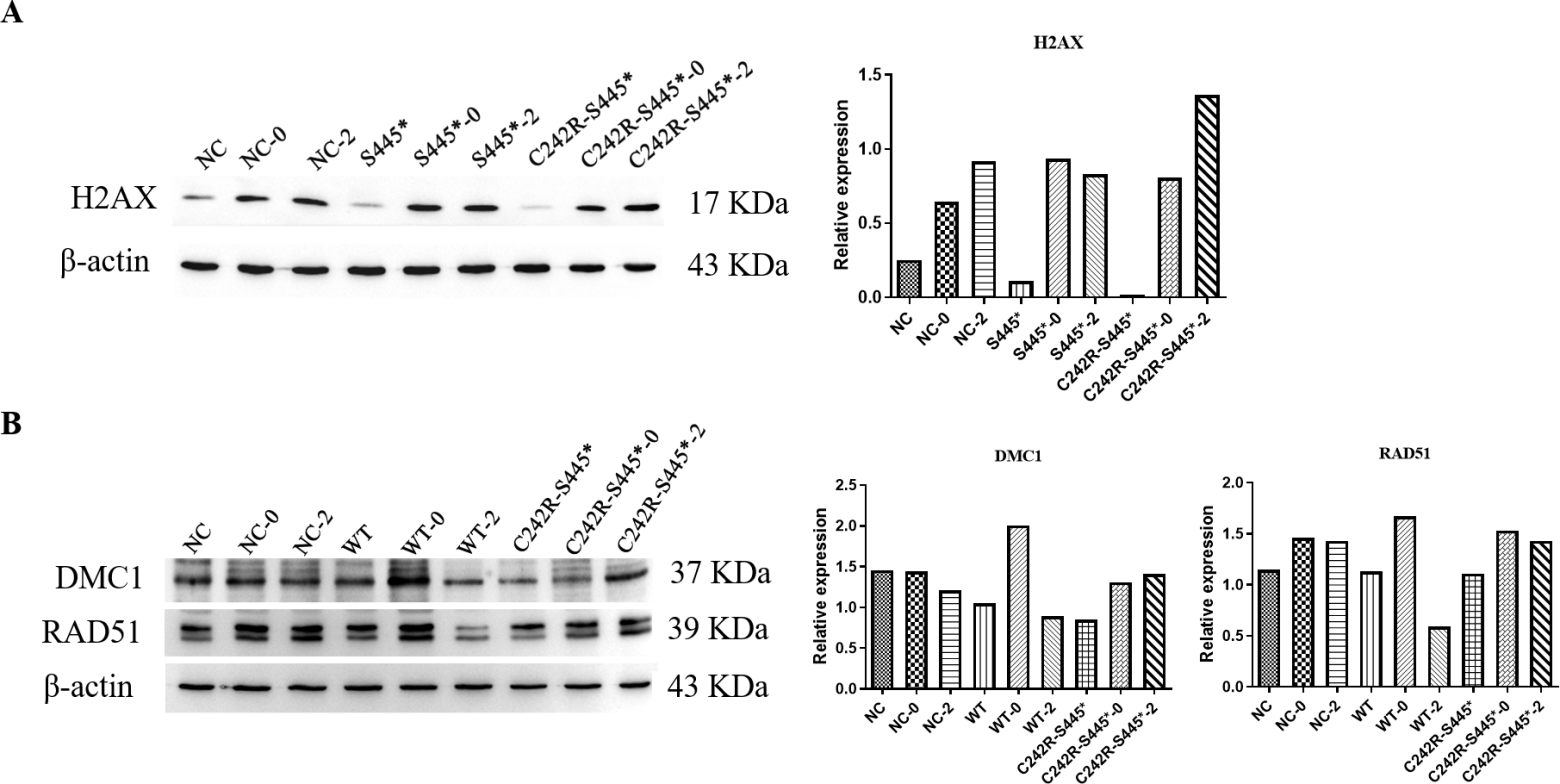
Effects of MCM8 mutation on DNA damage and repair. **(A)** Differences in the DNA damage repair response between the S445* mutation and the compound mutant C242R-S445* of MCM8. **(B)** Role of compound mutant MCM8 in the DMC1–RAD51 homologous recombination repair pathway. WT, overexpression of wild-type MCM8; -0, DNA damage; -2, DNA repair.

To assess cellular changes associated with the combined presence of the two variants *in vitro*, HeLa cells were transfected with an artificial cis double-mutant MCM8 overexpression construct harboring p.C242R and p.S445*. Following MMC treatment, all cells showed increased γ-H2AX levels, indicating activation of the DNA damage response and accumulation of DNA damage-associated signals. After 2 hours of recovery, γ-H2AX levels decreased in WT cells, suggesting resolution of DNA damage-associated signaling, but remained elevated in double-mutant construct. The C242R-S445* double mutant showed the strongest γ-H2AX induction post-MMC and paradoxical increase during recovery (**Figure 2A**). This indicates delayed resolution of DNA damage in cells expressing the artificial double-mutant MCM8 construct. We then analyzed the dynamics of key HR repair proteins. In WT cells, the recruitment of DMC1 and RAD51 was transient, peaking after damage and returning to near-baseline levels following recovery. This normal resolution of the HR machinery was abrogated in cells expressing the C242R-S445* mutant, in which elevated levels of both DMC1 and RAD51 persisted throughout the recovery period, with no significant decrease relative to the peak damage phase (**Figure 2B**). Based on these findings, which suggest delayed resolution of DNA damage-associated signaling and altered HR-related factor dynamics associated with double-mutant MCM8, the C242R-S445* construct was selected for subsequent functional investigations.

### 3.3 Cell-cycle and apoptosis changes associated with double-mutant MCM8

**Figure 3:**
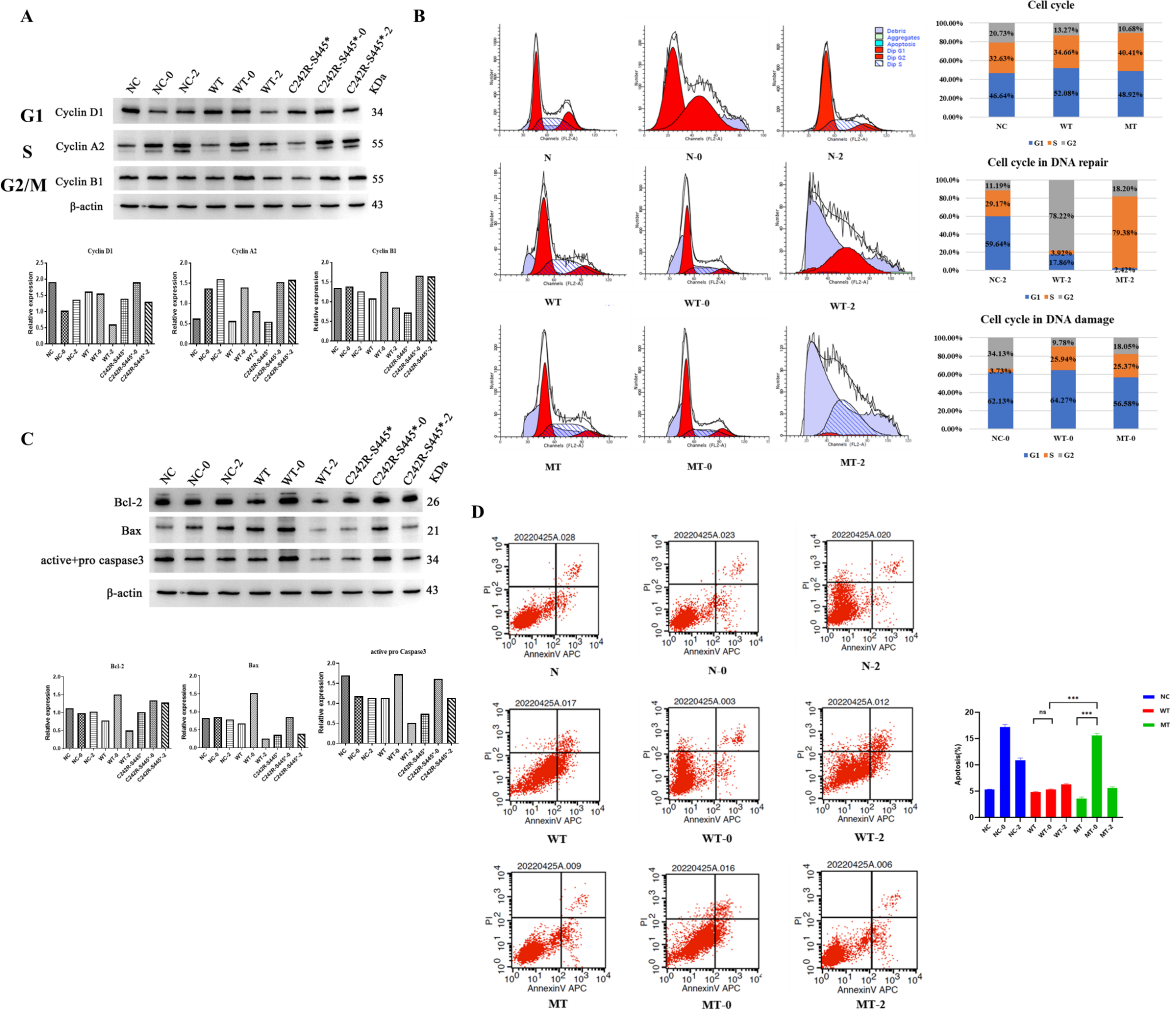
Effects of MCM8 mutation on the cell cycle and apoptosis. **(A)** Effects of a mutant MCM8 on cyclin expression during the DNA damage and repair phases. **(B)** Differential analysis of the effects of a mutant MCM8 on the cell cycle during the DNA damage and repair phases. NC: empty vector; WT: wild-type; MT: double-mutant C242R-S445* type; -0: DNA damage; -2: DNA repair. **(C)** Effect of mutant MCM8 on the expression of Bcl-2/Bax/caspase-3 apoptotic proteins during DNA damage repair. **(D)** A mutant MCM8 promotes apoptosis during DNA damage and repair. NC: empty vector; WT: wild-type MCM8; MT: double-mutant MCM8 (C242R-S445*); -0: DNA damage; -2: DNA repair. UR (upper right): Late apoptotic and necrotic cells (annexin V+/PI+). LL (lower left): Live cells (annexin V-/PI-). LR (lower right): Early apoptotic cells (annexin V+/PI-). *** p<0.001.

We further analyzed the effects of the double-mutant MCM8 on the cell cycle and apoptosis. After DNA damage induction, no obvious differences in the expression patterns of cyclin D1, cyclin A2, and cyclin B1 were observed between double-mutant cells (C242R-S445*-0) and wild-type cells (WT-0) (**Figure 3A**). During the recovery period, the expression of these three cyclins was downregulated in the wild-type cells (WT-2), whereas only cyclin D1 was downregulated in the double-mutant cells (C242R-S445*-2), and the expression of cyclin A2 and cyclin B1 remained unchanged (**Figure 3A**). Flow cytometry analysis revealed that the proportion of cells in the G1 phase increased in all groups after damage. After recovery, the proportion of G2 phase cells among the wild-type cells significantly increased, whereas the double-mutant cells exhibited a decrease in G1 phase cells and a significant increase in S phase cells (**Figure 3B**). Regarding apoptosis-related proteins, the expression of Bax, caspase-3, and Bcl-2 increased in both wild-type and double-mutant cells after damage induction (**Figure 3C**). After recovery, the expression of Bax in the wild-type cells significantly decreased, whereas the expression of Bcl-2 in the double-mutant cells did not decrease significantly, and the downregulation of expression of Bax and caspase-3 was delayed in the double-mutant cells (**Figure 3C**). Flow cytometry revealed that the total apoptosis rate of the double-mutant cells was significantly greater than that of the wild-type cells after damage; after recovery, the apoptosis rate of the double-mutant cells decreased, while the apoptosis rate of the wild-type cells did not significantly differ at different time points (**Figure 3D**). Together, these results indicate that expression of double-mutant MCM8 construct was associated with altered cell-cycle distribution and increased DNA damage-associated apoptotic signals in this overexpression model.

### 3.4 CoIP–MS analysis of proteins associated with wild-type and double-mutant MCM8

**Figure 4:**
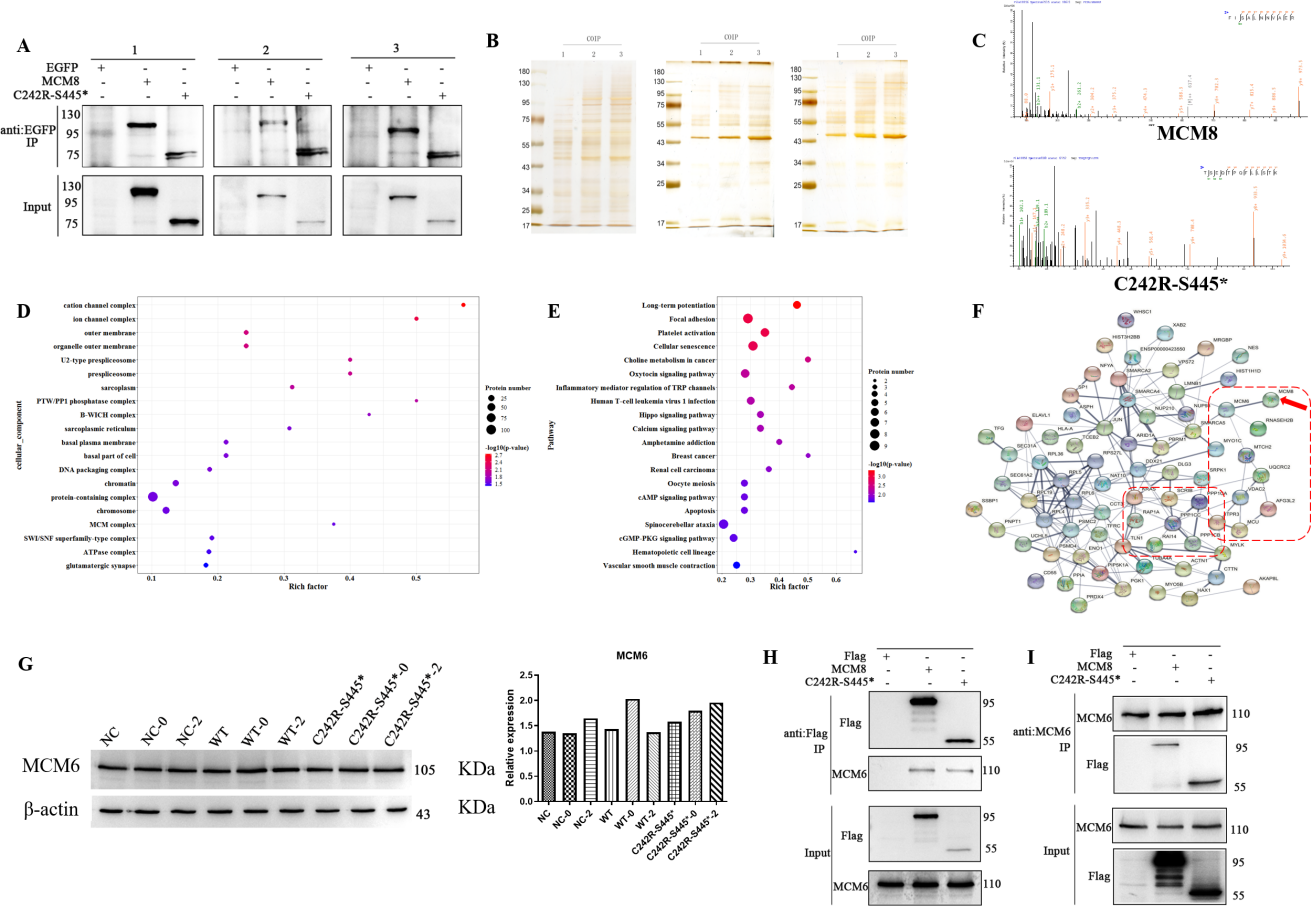
Detection of differentially interacting proteins of MCM8 by LC–MS/MS. **(A)** CoIP of wild-type and compound-mutant MCM8 in DNA repair. **(B)** Silver-stained gel images of wild-type and compound-mutant MCM8 in DNA repair. 1: empty vector; 2. wild-type MCM8; 3. double-mutant MCM8 (C242R-S445*). **(C)** Mass spectra of identified differentially expressed proteins. **(D)** Gene ontology (GO) analysis of differentially expressed proteins (DEPs) in both wild-type and double-mutant MCM8 cells on the basis of the CoIP/MS results. **(E)** Kyoto encyclopedia of genes and genomes (KEGG) analysis of differentially expressed proteins (DEPs) in both wild-type and double-mutant MCM8 cells on the basis of the CoIP/MS results. **(F)** String protein interaction network analysis. **(G)** Expression of MCM6 during the DNA damage repair period. **(H)** CoIP validation of candidate MCM8-interacting proteins using wild-type/mutant MCM8-flag as bait protein. **(I)** Reciprocal CoIP assay using MCM6 as the bait protein to validate its interaction with MCM8.

To identify candidate proteins associated with MCM8, we performed CoIP–MS analysis. Western blotting confirmed that the bait protein was successfully expressed and enriched in both wild-type and double-mutant MCM8 cells (**Figure 4A**), and silver staining revealed that the protein quantity met the requirements for mass spectrometry (**Figure 4B**). A total of 1768 proteins were identified by mass spectrometry. A total of 159 differentially enriched MCM8-associated proteins were identified between wild-type and double-mutant MCM8 (98 increased and 59 decreased) (**Table 1**). Notably, although MCM9 is the canonical binding partner of MCM8, it was not identified among these 159 differentially enriched proteins. Therefore, we did not detect evidence for a major change in MCM8–MCM9 co-enrichment under these experimental conditions. Further analysis revealed 14 common and 51 specific differentially enriched proteins (**Table 2**), among which RAI14, MCM6, and ETFB were detected across replicates (**Figure 4C**). GO functional annotation revealed that the differentially enriched MCM8-associated proteins were involved mainly in cell processes, metabolism, biological regulation, stress response, and protein complex assembly (**Table 3**). The significantly enriched terms closely related to the function of MCM8 included cation channel complex, ion channel complex, DNA packaging complex, protein-containing complex, and MCM complex (**Figure 4D**). KEGG pathway enrichment analysis showed that the differentially enriched MCM8-associated proteins were mainly related to cellular senescence, the calcium signaling pathway, oocyte meiosis, apoptosis, and endocrine resistance (**Figure 4E**). The protein interaction network analysis included 82 proteins, and the interaction score between MCM8 and MCM6 was the highest (0.948). RAI14 interacted with PPP1CC (0.532) and PPP1CB (0.538), whereas ETFB interacted with UQCRC2 (0.559) (**Figure 4F**, **Table 4**). Further validation revealed that MCM6 expression was upregulated in both wild-type and double-mutant cells during the DNA damage period (0 hours); during the repair period (2 hours), its expression decreased in wild-type cells but increased in double-mutant cells (**Figure 4G**). Bidirectional CoIP experiments supported the association between MCM8 and MCM6. In this assay, MCM6 showed greater co-immunoprecipitation with the double-mutant MCM8 construct than with wild-type MCM8 (**Figures 4H, I**).

**Table 1:** Mass spectrum results of common differential proteins.

Uniprot ID	Names of differentially expressed protein genes	The number of peptides identified for the protein	The number of unique peptides identified for the main protein	Sequence coverage [%]	Protein molecular weight [kDa]	Full-length protein	Q-value	Protein score	Mutation/wild trend
P06733	ENO1	20	20	40.6	47.168	434	0	187.3	DOWN
Q8N257	H2BU1	4	1	35.7	13.908	126	0.005364	2.0877	DOWN
Q9BYG3	NIFK	6	6	24.9	34.222	293	0	21.598	DOWN
**P13804**	**MCM8**	**11**	**10**	**17.9**	**93.696**	**840**	**0**	**30.145**	**DOWN**
**Q9UJA3Q9UJA3**	**MCM8-DM**	**3**	**3**	**8.1**	**49.788**	**444**	**0**	**30.145**	**UP**
Q9NUQ6	SPATS2L	3	3	5.7	61.728	558	0	9.7807	DOWN
P27816	MAP4	3	3	3.9	121	1152	0	10.046	DOWN
P38117	ETFB	10	10	35.7	27.843	255	0	31.802	UP
P13804	ETFA	6	6	27.6	35.079	333	0	20.439	UP
**Q14566**	**MCM6**	**11**	**11**	**16.3**	**92.888**	**821**	**0**	**81.51**	**UP**
P11498	PC	12	12	12.6	129.63	1178	0	78.231	UP
P0DP91	ERCC6	4	4	4.4	119.49	1061	0	9.2532	UP
**Q9P0K7**	**RAI14**	**2**	**2**	**3.4**	**110.04**	**980**	**0**	**7.6174**	**UP**
P51531	SMARCA2	4	3	2.8	181.28	1590	0	13.04	UP

The bold values represent proteins that we have verified in this study using Western blot and COIP experiments, as well as proteins associated with ovarian function.

**Table 2:** Mass spectrum results of unique differential proteins.

Uniprot ID	Names of differentially expressed protein genes	The number of peptides identified for the protein	The number of unique peptides identified for the main protein	Sequence coverage [%]	Protein molecular weight [kDa]	Full-length protein	Q-value	Protein score
Q96C19	EFHD2	16	15	57.5	26.697	240	0	110.62
Q9NYL9	TMOD3	19	19	51.7	39.594	352	0	90.013
Q71UM5	RPS27L	3	1	38.1	9.4771	84	0	7.2983
Q9UHB6	LIMA1	20	20	35.7	85.225	759	0	74.773
P38117	ETFB*	10	10	35.7	27.843	255	0	31.802
O00159	MYO1C	36	35	32.9	121.68	1063	0	130.16
P12814	ACTN1	24	16	32.3	103.06	892	0	62.295
P00558	PGK1	9	9	31.9	44.614	417	0	81.387
P62937	PPIA	7	7	30.9	18.012	165	0	41.061
O95400	CD2BP2	9	9	29	37.646	341	0	23.082
P13804	**ETFA**	6	6	27.6	35.079	333	0	20.439
Q96IX5	ATP5MK	2	2	27.6	6.4575	58	0	13.55
P49368	CCT3	10	10	26.4	60.533	545	0	128.74
Q15459	SF3A1	20	20	25.5	88.885	793	0	68.851
P16402	H1-3	9	1	24.4	22.35	221	0.0043	2.1922
O00165	HAX1	4	4	21.1	31.62	279	0	15.904
P42167	TMPO	6	2	20.5	50.67	454	0	3.4101
Q96EL2	**MRPS24**	3	3	18	19.015	167	0	4.2992
Q12797	ASPH	8	8	17.9	85.862	758	0	25.037
MCM8	MCM8	11	10	18.9	88.7	793	0	30.145
MCM8-DM	MCM8	3	3	8.1	49.788	444	0	30.145
P41091	EIF2S3	6	6	17.4	51.109	472	0	90.795
Q9BYD6	**MRPL1**	4	4	16.3	36.908	325	0	10.609
Q14566	**MCM6**	11	11	16.3	92.888	821	0	81.51
Q9NR30	DDX21	8	8	12.8	87.343	783	0	42.591
P23511	NFYA	2	2	12.1	36.876	347	0	19.957
Q96D15	RCN3	3	3	11.6	37.493	328	0	18.399
O15355	PPM1G	4	4	11.5	59.271	546	0	16.43
Q9H5V9	STEEP1	2	2	11.3	25.624	222	0	4.2999
P05186	ALPL	6	6	11.1	57.304	524	0	17.588
P45880	**VDAC2**	3	3	10.2	31.566	294	0	5.1019
Q5TBB1	RNASEH2B	2	2	9.9	35.138	312	0	16.555
Q8NE86	**MCU**	3	3	9.4	39.866	351	0	11.311
P84098	RPL19	2	2	9.2	23.466	196	0	5.6146
Q9Y580	RBM7	2	2	9	30.503	266	0	3.9184
Q9UN86	G3BP2	3	2	8.7	54.12	482	0	7.3792
Q13162	PRDX4	3	1	8.5	30.54	271	0	3.3313
Q9Y2X3	NOP58	3	3	7.4	59.578	529	0	6.7345
O60762	DPM1	2	2	6.9	29.634	260	0	4.6036
P31153	MAT2A	3	3	5.8	43.66	395	0	13.237
P22695	**UQCRC2**	2	2	5.7	48.442	453	0	34.246
Q99848	EBNA1BP2	2	2	5.6	34.852	306	0	4.4183
P0DP91	ERCC6	4	4	4.4	119.49	1061	0	9.2532
P16278	GLB1	2	2	4.3	76.074	677	0	6.9092
P48681	NES	6	6	3.5	177.44	1621	0	15.075
Q9ULX6	AKAP8L	2	2	3.4	71.639	646	0	5.149
Q9P0K7	**RAI14**	2	2	3.4	110.04	980	0	7.6174
P51531	SMARCA2	4	3	2.8	181.28	1590	0	13.04
Q9Y490	TLN1	4	4	2.2	269.76	2541	0	8.2563
Q9ULV0	MYO5B	3	2	2.1	213.67	1848	0	7.951
O96028	NSD2	3	3	2.1	152.26	1365	0	12.799

**Table 3:** GO secondary classification results.

Ontology	name	Go ID	Count
biological_process	**cellular process**	GO:0050875	154
biological_process	**metabolic process**	GO:0044710	125
biological_process	**biological regulation**	GO:0065007	101
biological_process	response to stimulus	GO:0051869	71
biological_process	multicellular organismal process	GO:0050874	63
biological_process	localization	GO:1902578	54
biological_process	**reproduction**	GO:0050876	11
biological_process	**reproductive process**	GO:0044702	11
cellular_component	cellular anatomical entity	GO:0110165	158
cellular_component	**protein-containing complex**	GO:0043234	105
molecular_function	binding	GO:0005488	151
molecular_function	catalytic activity	GO:0003824	57
molecular_function	**ATP-dependent activity**	GO:0140657	18

**Table 4:** Protein interaction PPi information.

accession_node1	accession_node2	#node1	node2	combined_score
Q14573	Q8NE86	ITPR3	MCU	0.898
Q14573	P45880	ITPR3	VDAC2	0.745
**Q14566**	**MCM8;MCM8-amphimutation**	**MCM6**	**MCM8**	**0.948**
Q8NE86	P45880	MCU	VDAC2	0.625
P22695	P45880	UQCRC2	VDAC2	0.846
Q14566	Q9Y6C9	MCM6	MTCH2	0.434
P20700	Q14566	LMNB1	MCM6	0.58
Q14566	O60264	MCM6	SMARCA5	0.69
Q14566	Q5TBB1	MCM6	RNASEH2B	0.429
**P62140**	**Q9P0K7**	**PPP1CB**	**RAI14**	**0.538**
**P36873**	**Q9P0K7**	**PPP1CC**	**RAI14**	**0.532**
**P38117**	**P22695**	**ETFB**	**UQCRC2**	**0.559**
Q14566	Q15459	MCM6	SF3A1	0.45

The bold values represent proteins that we have verified in this study using Western blot and COIP experiments, as well as proteins associated with ovarian function.

### 3.5 Cut&Tag–mRNA-seq analysis of MCM8-associated chromatin-binding and transcriptomic changes

**Figure 5:**
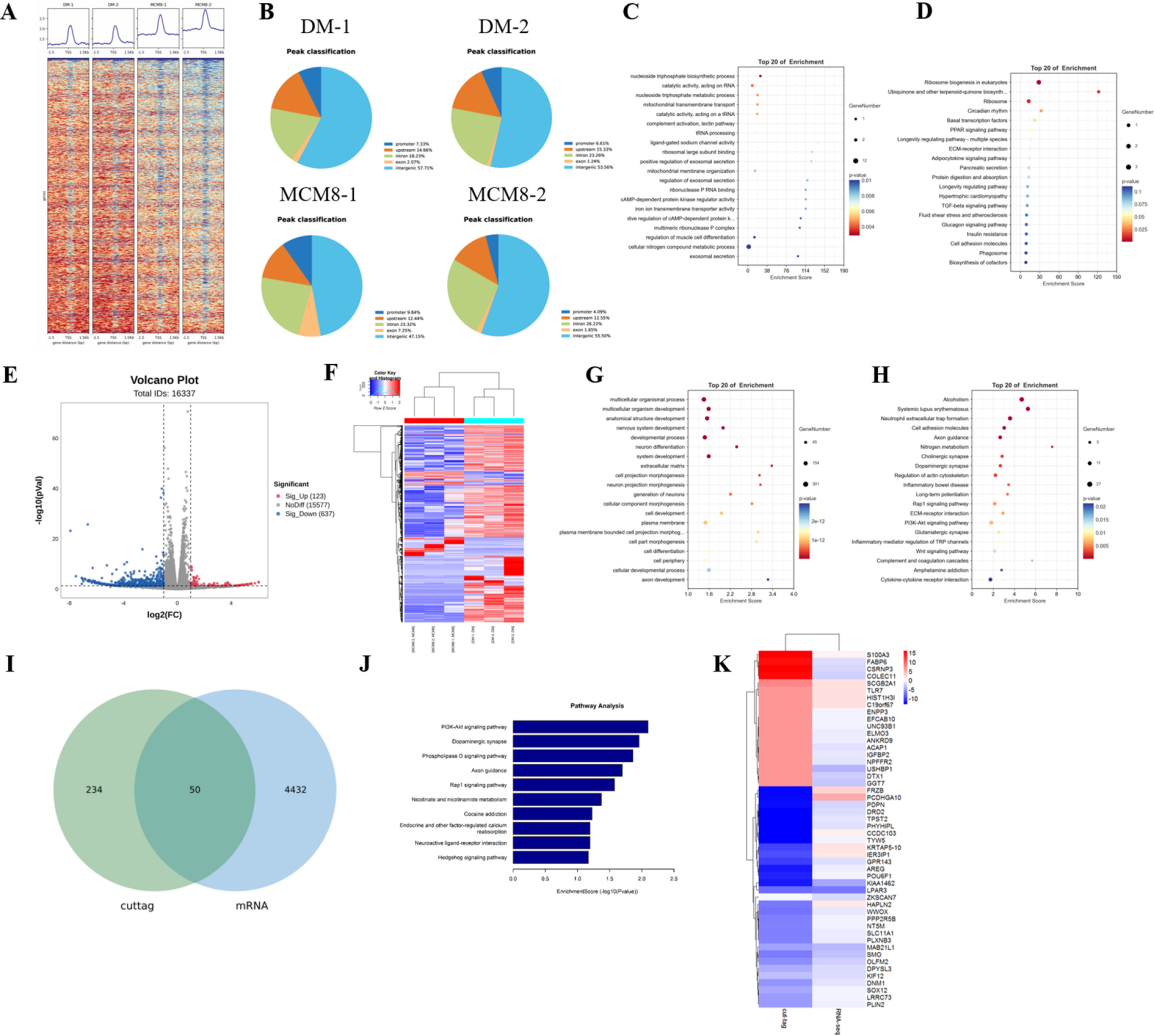
Identification of genes associated with altered MCM8 chromatin binding and differential expression by Cut&Tag–mRNA-seq analysis. **(A)** Genome-wide chromatin binding profile of MCM8. **(B)** Genomic mapping analysis of MCM8 binding sites. **(C)** Gene Ontology (GO) analysis of differentially expressed genes (DEGs) in both wild-type and double-mutant MCM8 cells on basis of the Cut&Tag assay results. **(D)** Kyoto encyclopedia of genes and genomes (KEGG) analysis of differentially expressed genes (DEGs) in both wild-type and double-mutant MCM8 cells on the basis of the Cut&Tag assay results. **(E)** Volcano plot showing differentially expressed genes (DEGs) between wild-type and double-mutant MCM8 cells based on RNA-seq analysis. **(F)** Heatmap showing differentially expressed genes (DEGs) between wild-type and double-mutant MCM8 cells based on RNA-seq analysis. **(G)** Gene Ontology (GO) analysis of differentially expressed genes (DEGs) in both wild-type and double-mutant MCM8 cells on the basis of the RNA-Seq results. **(H)** Kyoto encyclopedia of genes and genomes (KEGG) analysis of differentially expressed genes (DEGs) in both wild-type and double-mutant MCM8 cells on the basis of the RNA-Seq results. **(I)** Venn diagram of key genes in the Cut&Tag–RNA-seq cross-linking analysis. **(J)** Mutant MCM8 is enriched in POI-related signaling pathways according to Cut&Tag–RNA-seq cross-linking analysis. **(K)** Mutant MCM8 is enriched in POI-related genes according to Cut&Tag–RNA-seq cross-linking analysis. (n=3 biological replicates).

To elucidate the function of double-mutant MCM8 in genomic binding, we performed the Cut&Tag assay and RNA-seq analyses on cells carrying the wild-type plasmid, the double-mutant *MCM8* plasmid, or empty vector during the DNA repair period. The Cut&Tag assay mapped the chromatin binding profile of MCM8 across the entire genome (**Figure 5A**), with binding peaks distributed mainly in intergenic regions (**Figure 5B**). The double-mutant MCM8 had 7379 differential binding sites, among which 2074 showed increased binding and 5305 showed decreased binding. GO functional analysis indicated that the differentially genes were enriched mainly in mitochondrial transmembrane transport, mitochondrial membrane organization, and the cellular nitrogen compound metabolic process (**Figure 5C**). KEGG enrichment analysis revealed that the differentially genes bound to MCM8 were enriched mainly in ribosome biogenesis in eukaryotes, ubiquinone and other terpenoid-quinone biosynthesis, and the PPAR signaling pathway (**Figure 5D**). RNA-seq analysis revealed 760 differentially expressed mRNAs, including 637 upregulated and 123 downregulated mRNAs (**Figures 5E, F**). GO functional analysis indicated that the differentially expressed genes were enriched mainly in multicellular organism development, the development process, system development, cell development and the cellular developmental process (**Figure 5G**). KEGG enrichment analysis of the differentially expressed genes showed enrichment mainly in cell adhesion molecules and the PI3K–Akt signaling pathway (**Figure 5H**). By integrating the Cut&Tag binding sites and differentially expressed genes identified by RNA-seq, we identified 50 candidate genes showing both altered MCM8-associated binding and altered transcript levels (**Figure 5I**). Further combined analyses highlighted transcriptomic changes involving genes related to the PI3K/AKT signaling pathway in the double-mutant group (**Figure 5J**), and PPP2R5B expression increased in double-mutant group (**Figure 5K**). To verify the differentially expressed candidate genes of the PI3K/AKT signaling pathway, the relative mRNA expression levels were assessed using qRT–PCR. As shown in **Supplementary Figure 1**, the mRNA levels of PTEN, PIK3AP1, PPP2R5B, and CREB5 were significantly higher in double-mutant MCM8 group (DM) than in the control. These results indicate that double-mutant MCM8 was associated with chromatin-binding-related and transcriptomic changes involving candidate genes related to PI3K–AKT signaling.

## 4 Discussion

In our previous clinical study, we reported a Chinese family that presented with pediatric-onset POI harboring two compound heterozygous variants (NM_001281522.1: c.724T>C [p.Cys242Arg] and c.1334C>A [p.Ser445*]) in the *MCM8* gene (27). Clinically, the compound heterozygous variants presented as progressive ovarian atrophy in two affected sisters, marked by follicular depletion, hypergonadotropic hypogonadism, and premature apoptosis—features consistent with autosomal recessive POI, as previously documented (23, 27–30). Notably, the proband’s younger sister had normal uterine and ovarian volumes and FSH levels at 6 years and 7 months of age. However, at the age of 7 years and 10 months, ultrasound revealed that the bilateral ovaries gradually progressed from a normal size to an unclear display, accompanied by a decrease in the FSH level, and at follow-up at 11 years of age, the FSH level progressively manifested as hypergonadotrophic hypogonadism. These data indicated the age-dependent penetrance of MCM8-related defects and reinforced the importance of early genetic screening in families with a history of POI. Building upon these clinical findings, in the present study, we aimed to provide model-based *in vitro* observations regarding cellular changes associated with double-mutant MCM8. Our integrated cellular and multi-omics analyses using an artificial cis double-mutant overexpression model suggest that the co-occurrence of p.C242R and p.S445* in the same MCM8 construct is associated with delayed DNA damage resolution, altered recruitment of HR-related factors, changes in MCM8-associated protein interactions, and transcriptomic alterations. These *in vitro* observations may generate hypotheses regarding cellular processes potentially affected by MCM8 dysfunction, but they do not directly establish the physiological pathogenesis of POI in patients carrying compound heterozygous variants.

The canonical cellular function of MCM8, which functions primarily as a heterodimer with MCM9, is to act as a DNA helicase that unwinds double-stranded DNA during homologous recombination. This process is indispensable for repairing DNA double-strand breaks (DSBs) and maintaining meiotic fidelity in gonadal tissues (18, 22). This observations are consistent with previous studies showing that MCM8 deficiency in mice causes defects in primordial germ cell proliferation and meiosis, ultimately leading to premature ovarian failure (31, 32). Structural modeling and functional assays suggested that the p.S445* truncation variant in the AAA domain may induce greater structural perturbation than the p.C242R variant in the zinc finger domain, which is consistent with a stronger impact on the DNA damage response in our model. Our *in vitro* assays revealed that cells expressing double-mutant MCM8 exhibited delayed resolution of DNA damage response. Persistent γ-H2AX accumulation suggests impaired or delayed DNA damage repair, while altered recruitment of recombination-associated factors, including RAD51 and DMC1, indicates disruption of homologous recombination-related processes. This unresolved DNA damage may contribute to S-phase accumulation and cell apoptosis.

While MCM8 is canonically known to form a functional heterodimer with MCM9, our CoIP-MS/MS screening did not identify MCM9 as a significantly altered interactor between the wild-type and double-mutant MCM8. Because the canonical MCM8-MCM9 interaction was not significantly disrupted according to our differential screening criteria, our interactome data unexpectedly directed our focus toward a profound shift in the interaction between the mutant MCM8 and MCM6. MCM6 is an essential core subunit of the MCM2–7 replicative helicase complex, which is strictly required for DNA replication origin licensing and replication fork progression (33). The altered MCM6 association observed in our interactome data led us to hypothesize that mutant MCM8 may affect MCM2–7 complex dynamics indirectly or through aberrant protein association. Such altered interactions could potentially affect replication-associated processes and contribute to replication stress in this overexpression system. Consistent with this possibility, changes in apoptosis-related markers were observed. However, we acknowledge that the precise biophysical consequences of this aberrant MCM8–MCM6 binding remain highly speculative. In the current experimental design, no direct causal link between this disrupted MCM8-MCM6 interaction and the observed apoptotic phenotype has been strictly established. Whether altered MCM6 association directly contributes to replication stress and apoptosis in these cells, or instead represents a parallel consequence of the altered conformation of the double-mutant protein, remains unknown. Moreover, the lack of targeted validation of the MCM8–MCM9 interaction in the present study limits a comprehensive interpretation of the homologous recombination-related phenotype.

Beyond its role in DNA repair, our Cut&Tag and RNA-seq analyses revealed that the *MCM8* mutations were associated with altered chromatin binding profiles and transcriptomic changes. It is crucial to clarify that our data do not establish MCM8 as a direct, classical transcription factor. Rather, we hypothesize that the altered chromatin occupancy of the mutant MCM8 protein may indirectly influence transcriptomic regulation. Notably, the candidate genes identified in our multi-omics intersection analysis, including PIK3AP1, CREB5, PPP2R5B, and PTEN, were related to the PI3K/AKT signaling pathway. The PI3K/AKT cascade is an important regulator of primordial follicle activation, survival, and metabolic homeostasis.

Despite providing valuable functional annotations for these pathogenic variants, our findings must be interpreted with caution because of several substantial methodological limitations. First, our cellular model relied on HeLa cells, a highly aneuploid cervical cancer cell line whose baseline transcriptomic networks, cell cycle checkpoints, and chromatin landscapes differ from those of primary human ovarian granulosa cells. Extrapolating these *in vitro* findings to human ovarian biology is inherently limited. Second, the mutant constructs were transiently overexpressed in the presence of endogenous wild-type MCM8, which may introduce spurious protein–protein interactions because of altered stoichiometry. Third, our *in vitro* 33experimental design used a single expression construct harboring both variants (in cis), which inherently generates an artificial double-mutant protein. We acknowledge that this experimental strategy does not reproduce the endogenous compound heterozygous configuration (in trans) observed on patients’ homologous chromosomes. We recognize that accurately modeling true biallelic compound heterozygosity within standard *in vitro* cell models remains a technical challenge. While co-transfecting two separate single-mutant plasmids was considered as an alternative to mimic the trans configuration, such transient expression systems are limited by stochastic plasmid uptake. Due to these current technical limitations, such systems cannot reliably reproduce physiological 1:1 stoichiometric expression of both mutant alleles at the single-cell level, nor can they replicate endogenous chromosomal regulatory mechanisms. Therefore, our *in vitro* system does not model the patient’s precise physiological phenotype but rather serves as a related but distinct artificial model to explore the combined functional consequences of these specific variant combinations in an overexpression setting. Fourth, the lack of assessment of the MCM8–MCM9 heterodimer leaves a critical gap in our mechanistic understanding. Finally, comparing apoptotic markers between patient-derived streak gonads (which are highly fibrotic tissue devoid of functional follicles) and functional normal ovarian margins is subject to inherent biological bias.

**Figure 6:**
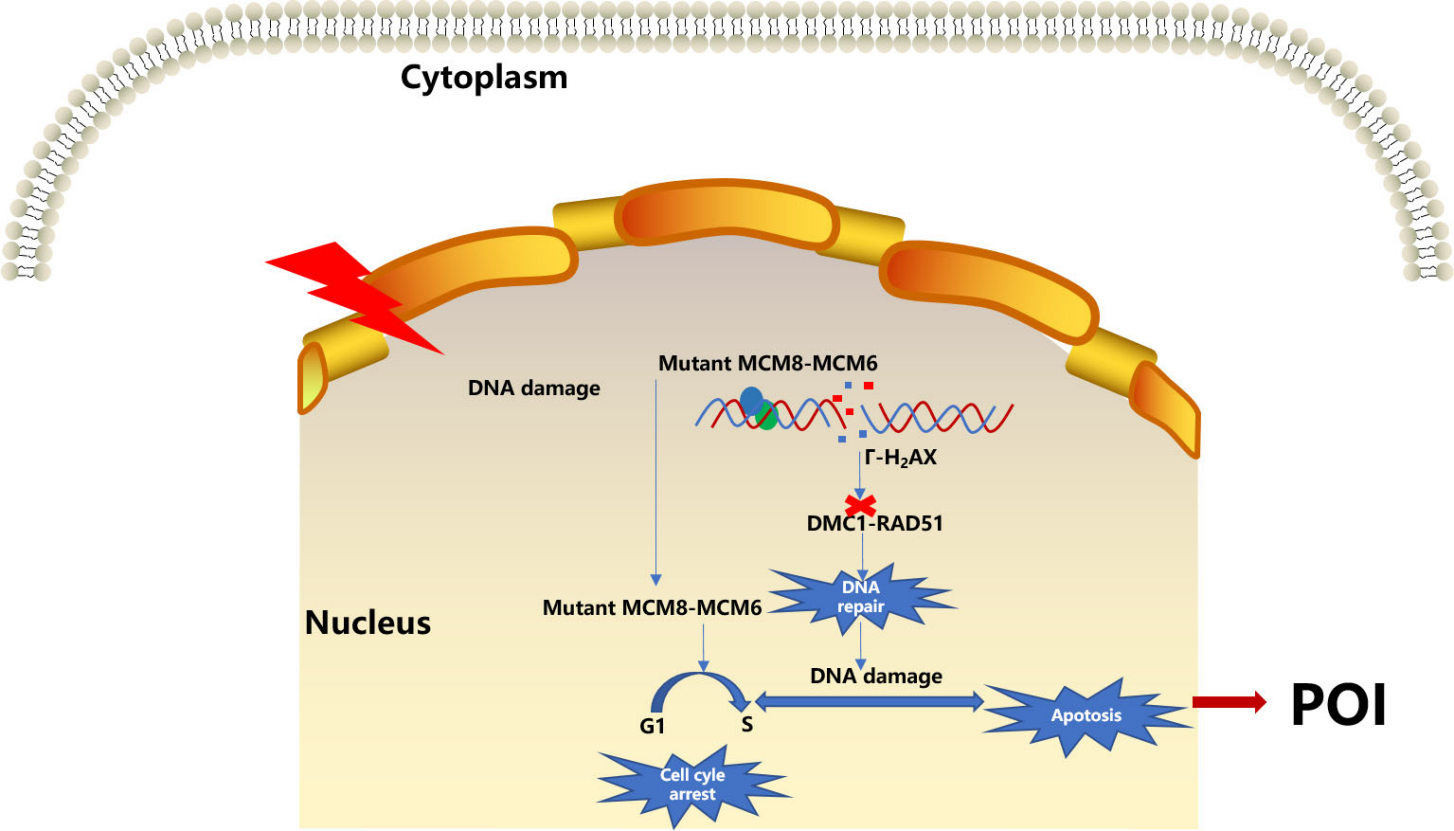
Schematic model of cellular abnormalities associated with expression of double-mutant MCM8 construct *in vitro*. Expression of the artificial cis double-mutant MCM8 construct harboring p.C242R and p.S445* was associated with altered MCM8–MCM6 interaction, persistent γ-H2AX accumulation, impaired recruitment of homologous recombination-related factors, including DMC1 and RAD51, delayed DNA damage resolution, S-phase accumulation/cell-cycle arrest, and increased apoptotic signaling *in vitro*. These cellular abnormalities may provide model-based clues regarding the potential effects of MCM8 dysfunction on DNA repair and cell survival.

In conclusion, this study reports the clinical association of pediatric-onset POI with compound heterozygous MCM8 variants, p.C242R and p.S445*, and provides an *in vitro* functional characterization using an artificial double-mutant MCM8 overexpression construct. Rather than defining the physiological pathogenesis of the patients, our findings show that expression of this double-mutant construct is associated with several cellular abnormalities *in vitro*. First, cells expressing the double-mutant construct showed delayed resolution of DNA damage, persistent γ-H2AX accumulation, and altered recruitment of HR-related factors, together with S-phase accumulation. Second, altered interaction between double-mutant MCM8 and MCM6 may reflect changes in replication-associated protein complexes and may be related to replication stress in this overexpression system. Third, integrated Cut&Tag and RNA-seq analyses identified MCM8-associated chromatin-binding changes and transcriptomic alterations involving candidate genes in PI3K/AKT signaling pathway (**Figure 6**). Collectively, these findings provide model-based cellular insights that may help generate hypotheses regarding how MCM8 dysfunction affects DNA damage repair, cell-cycle progression and apoptosis. To validate these molecular mechanisms and accurately model compound heterozygosity in a physiological context, future studies employing more physiological models, such as biallelic CRISPR/Cas9-mediated knock-in systems or granulosa-like cells, will be required to validate the physiological relevance of these findings.

## Data Availability

The raw sequence data generated in this study have been deposited in the Genome Sequence Archive (34) in National Genomics Data Center (35), China National Center for Bioinformation / Beijing Institute of Genomics, Chinese Academy of Sciences (GSA-Human: HRA016491) that are publicly accessible at https://ngdc.cncb.ac.cn/gsa-human. The data that support the findings of this study are available from the corresponding authors upon reasonable request.

## Supplementary material

Supplementary Figure 1 Cut&Tag-seq and RNA-seq cross-linking analysis of the expression of genes related to the PI3K/AKT pathway in MCM8 wild-type and mutant cells during the DNA repair period. Relative mRNA levels of genes involved in the PI3K/AKT signaling pathway, including PTEN, PPP2R5B, LPAR3, CREB5, PIK3AP1, PIK3R3, and AKT3. ****P* < 0.001, **P* < 0.05, ns *P* > 0.05, n=3 biological replicates.

Supplementary Table 1 Antibodies used.

Supplementary Table 2 Sequences of qPCR primers.
