# Supplementary material for: MCM8 variants in two patients with primary ovarian insufficiency: clinical findings and in vitro defective DNA repair revealed by an MCM8 double-mutant construct
Source: Front Endocrinol (Lausanne). 2026 Jun 17;17:1807887. doi: 10.3389/fendo.2026.1807887 (PMC13318747; doi:10.3389/fendo.2026.1807887)
Supplement: Supplementary Figure 1 — Cut&Tag-seq and RNA-seq cross-linking analysis of the expression of genes related to the PI3K/AKT pathway in MCM8 wild-type and mutant cells during the DNA repair period. Relative mRNA levels of genes involved in the PI3K/AKT signaling pathway, including PTEN, PPP2R5B, LPAR3, CREB5, PIK3AP1, PIK3R3, and AKT3. ***P < 0.001, *P < 0.05, ns P > 0.05, n=3 biological replicates. [file SupplementaryFile1.doc]

**Supplementary materials**

Table 1 Antibodies Used

| **Target Protein** | **Host Species** | **Supplier** | **Catalog Number** | **Dilution** | **Application** |
| --- | --- | --- | --- | --- | --- |
| MCM8 | Rabbit | NOVUS | NBP2-55276 | 1:100 | IHC/IF |
| H2AX | Rabbit | CST | #2595 | 1:1000 | WB |
| β-actin | mouse | fitgene | FI01103 | 1:5000 | WB |
| DMC1 | Rabbit | Proteintech | 13714-1-AP | 1:1000 | WB |
| RAD51 | Rabbit | Proteintech | 14961-1-AP | 1:1000 | WB |
| Cyclin D1 | Rabbit | ABclonal | A19038 | 1:5000 | WB |
| Cyclin A2 | Rabbit | ABclonal | A19036 | 1:5000 | WB |
| Cyclin B1 | Rabbit | ABclonal | A19037 | 1:1000 | WB |
| Bcl-2 | Rabbit | ABclonal | A19693 | 1:1000 | WB |
| Bax | Rabbit | ABclonal | A19684 | 1:1000 | WB |
| active + pro Caspase 3 | Rabbit | ABclonal | A19654 | 1:1000 | WB |
| MCM6 | Rabbit | Proteintech | 13347-2-AP | 1:2000/1:1000 | WB/IP |
| Flag | mouse | ABclonal | AE005 | 1:5000 | IP |
| GFP | mouse | Santa Cruz Biotechnology | sc-9996 | 1:1000 | IP |
| HRP-conjugated Goat anti-Rabbit IgG (H+L) | Goat | ABclonal | AS014 | 1:5000 | WB |
| HRP-conjugated Goat anti-Mouse IgG (H+L) | Goat | ABclonal | AS003 | 1:5000 | WB |
| Cy3-conjugated Goat anti-Rabbit IgG (H+L) | Goat | ABclonal | AS007 | 1:500 | IF |

**Table 2 Sequences of qPCR primers.**

| **Gene** | **Species** | **Forward primer (5’-3’)** | **Reverse primer (5’-3’)** |
| --- | --- | --- | --- |
| *PTEN* | human | GACCAGAGACAAAAAGGGAGTA | ACAAACTGAGGATTGCAAGTTC |
| *LPAR3* | human | TAATTTAGCTGCTGCCGATTTC | CTTTTTGGTCAGGTTGCTATGG |
| *PPP2R5B* | human | AACGAGCTGGTGGAGTGTGT | ATGCGGATGATGTCTGGGTA |
| *β-ACTIN* | human | GTGGATCAGCAAGCAGGAGT | AAAGCCATGCCAATCTCATC |
| *PIK3R3* | human | GCCCTATTCGACAGAACTGATA | TTTGGAACTGCTGAAGTCATTG |
| *PIK3AP1* | human | AAGTAGCACAAGTAACCGCTC | CCTCAGGGACTTCATTATCCTC |
| *CREB5* | human | GCAACAAGTCATCCAGCATAAT | AAGAATCGGATTCAGGTCTGTT |
| *AKT3* | human | GCAGAGGCAAGAAGAGGAGA | CCACTTGCCTTCTCTCGAAC |

**Supplementary Figure 1**


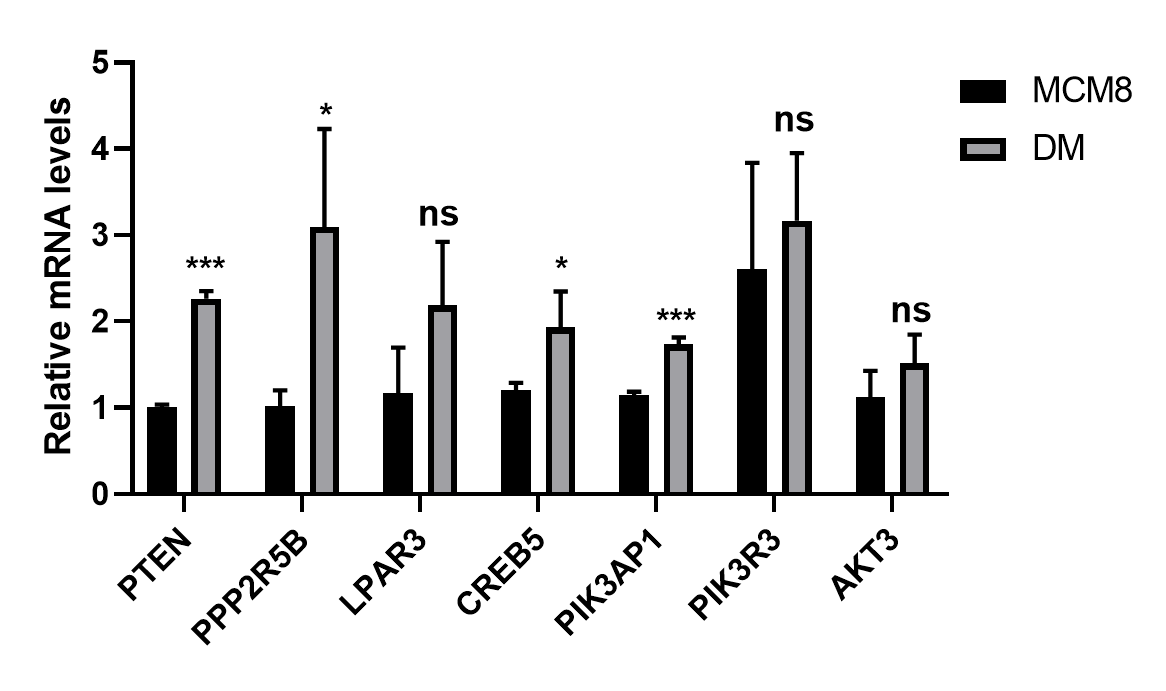


**Supplementary Figure 1**. Cut&tag-seq and mRNA-seq cross-linking analysis of the expression of genes related to the PI3K/AKT pathway in MCM8 wild-type and mutant cells during the DNA repair period. *** *P* < 0.001, * *P* < 0.05, ns *P* > 0.05.
